# The genetic tale of a recovering lion population (*Panthera leo*) in the Savé Valley region (Zimbabwe): A better understanding of the history and managing the future

**DOI:** 10.1371/journal.pone.0190369

**Published:** 2018-02-07

**Authors:** Laura Tensen, Rosemary J. Groom, Joy Khuzwayo, Bettine Jansen van Vuuren

**Affiliations:** 1 Centre for Ecological Genomics and Wildlife Conservation, Department of Zoology, University of Johannesburg, Johannesburg, South Africa; 2 African Wildlife Conservation Fund, Chishakwe Ranch, Savé Valley Conservancy, Zimbabwe; University of Innsbruck, AUSTRIA

## Abstract

The rapid decline of the African lion (*Panthera leo*) has raised conservation concerns. In the Savé Valley Conservancy (SVC), in the Lowveld of Zimbabwe, lions were presumably reduced to approximately 5 to 10 individuals. After ten lions were reintroduced in 2005, the population has recovered to over 200 lions in 2016. Although the increase of lions in the SVC seems promising, a question remains whether the population is genetically viable, considering their small founding population. In this study, we document the genetic diversity in the SVC lion population using both mitochondrial and nuclear genetic markers, and compare our results to literature from other lion populations across Africa. We also tested whether genetic diversity is spatially structured between lion populations residing on several reserves in the Lowveld of Zimbabwe. A total of 42 lions were genotyped successfully for 11 microsatellite loci. We confirmed that the loss of allelic richness (probably resulting from genetic drift and small number of founders) has resulted in low genetic diversity and inbreeding. The SVC lion population was also found to be genetically differentiated from surrounding population, as a result of genetic drift and restricted natural dispersal due to anthropogenic barriers. From a conservation perspective, it is important to avoid further loss of genetic variability in the SVC lion population and maintain evolutionary potential required for future survival. Genetic restoration through the introduction of unrelated individuals is recommended, as this will increase genetic heterozygosity and improve survival and reproductive fitness in populations.

## Introduction

In an increasingly human dominated and fragmented landscape, biodiversity has a changing face. Not surprisingly, an increasing number of species are adversely affected and show signs of negative population growth. A recent study by Ceballos and co-workers [1] reported that almost half of the 177 mammal species studied have so far lost more than 80% of their natural ranges during the Anthropocene. Although the African continent is amongst the most biodiverse, the highest percentage of habitat loss occurred here [1]. Arguably, species most affected by anthropogenic pressures are those in direct conflict with humans, such as apex predators. A case in hand is Africa's top predator, the lion (*Panthera leo*). The main threats to lion populations include habitat loss, persecutions (often to protect livestock), the depletion of the natural prey base, and, in some cases, poorly managed trophy hunting [2, 3].

The rapid decline of the African lion has raised conservation concerns. Population size has decreased from roughly 75,000 individuals to 32,000 individuals in only two decades [4–6], with a range reduction of approximately 25% of original savannah habitat [4]. Currently, only ten population strongholds remain in East and Southern Africa; strongholds are defined here as areas that are protected, support at least 500 individuals, and where the resident lion population is stable or increasing in size [4].

Southern African lion populations are declining at lower rates than elsewhere in Africa, due to lower human population densities, healthier prey populations, more support (including financial) for conservation management, and the more common use of park fences [6]. Lion densities are higher in fenced reserves (compared with unfenced areas) due to reduced bushmeat hunting and human-wildlife conflict [3]. Although trophy hunting of lions is still common in Southern Africa, usually a percentage of the funds generated through this practise is channelled into maintenance of nature reserves and wildlife parks, thereby benefitting lion conservation [7, 8].

**Fig 1:**
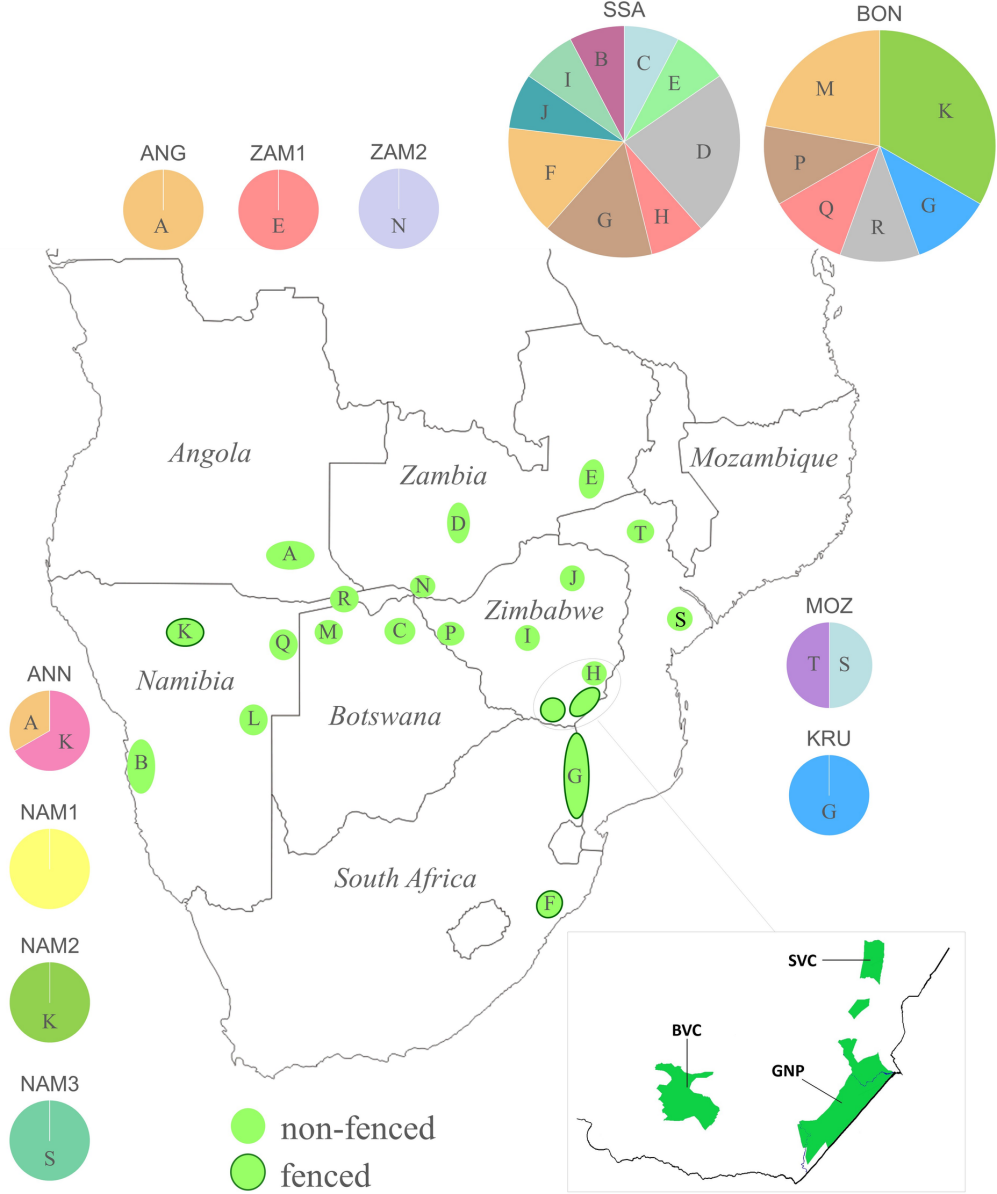
A map of Africa, showing the haplotypes used during our study [42, 43, 44, 45]. Our sampling sites, in the Zimbabwean part of the Greater Limpopo Transfrontier Conservation Area, are highlighted: Savé Valley Conservancy (SVC), Bubye Valley Conservancy (BVC), and Gonarezhou National Park (GNP).

Zimbabwe still supports relatively stable (in some areas even increasing) lion populations, as the probability of extinction is considered low in the majority of areas that contain lions [5]. Although trophy hunting on privately owned land has been an important source of income that has enabled the protection of wildlife during political and economic instability [9, 10], this is not always practised in a responsible manner, as was shown to be the case in Hwange National Park [11]. Specifically, lion conservation will only benefit from trophy hunting if the offtake is sustainable [12], which is recommended to be 1 male lion (≥ 5 years of age) per 2,000 km^2^ [13]. In Bubye Valley Conservancy (hereafter BVC), situated in the Lowveld of Zimbabwe (Fig 1), trophy hunting is controlled by strict quotas, and other negative anthropogenic impacts on the local lion population are mitigated by fences and anti-poaching patrols. This privately owned reserve reintroduced 13 lions with Namibian origin in 1999, and currently holds a population of between 500 and 550 lions, with an extremely high population density (16 lions per 100 km^2^) [14, 15]. Since the reintroduction, young males have occasionally entered the park from surrounding areas, such as Gonarezhou National Park. Gonarezhou National Park (hereafter GNP), is also fenced and even though wildlife populations have been fluctuating, there has always been a resident lion population (recent estimates are approximately 250 individuals [16]). The nearby Savé Valley Conservancy (hereafter SVC) is not completely fenced, and resident wildlife populations have suffered from illegal bushmeat hunting and poaching [17]. Prior to the establishment of the SVC as a wildlife conservancy in 1992, when the area was still used for cattle ranching, lion numbers were reduced to approximately 5 to 10 individuals. Subsequent to the introduction of ten lions in 2005, the population recovered to over 200 lions in 2016 [18]. The origin of the reintroduced lions is, however, unclear as no records exist to clarify this.

Although the increase of lions in the SVC and BVC seems promising, a question remains whether the populations are genetically viable, considering their small founding population. Furthermore, to ensure a genetically healthy lion population, Björklund [19] recommends that a minimum of 50 prides be maintained, with no restrictions to dispersal. These criteria are currently not met in the Zimbabwean Lowveld, due to habitat loss and restricted dispersal between reserves [10]. Genetic depletion, because of inbreeding and genetic drift in small populations, can have serious negative consequences for wildlife populations [20, 21]. This became apparent in the Hluhluwe-Imfolozi Park, South Africa, where low genetic diversity and inbreeding depression in an isolated lion population led to high cub mortality, poor physical condition of adults, and reduced immuno-competence [22]. Genetic variation was restored in this population through the translocation of unrelated males into this closed population [23], with a subsequent improvement in the reproductive success. Another striking example has been a highly inbred population of Florida panthers *Puma concolor*, where the population increased by three-fold after heterozygosity levels recovered subsequent to the introduction of eight unrelated females [24].

In this study, we address two main questions. First, we document the genetic diversity in the lion populations sampled during this study (i.e. SVC, BVC and GNP) using neutral genetic markers, both mitochondrial and nuclear, and compare our results to literature from other lion populations across Africa. We use the term ‘population’ as the localized, breeding group of lions in each reserve. We pay special attention to the number of haplotypes (indicative of the number of founding lineages), as well as allelic richness and heterozygosity (indicative of recent population trends). Although only adaptive diversity is directly related to genetic potential (i.e. presence of sufficient diversity to allow for future adaption) [20, 25], neutral markers (such as the microsatellites loci used during our study) are better suited to compare and assess demography and other parameters such as population bottlenecks, genetic diversity and inbreeding in the absence of natural selection [26].

Secondly, we tested whether similar or closely related genetic lineages are present in the different conserved areas. As genetic diversity is a key component of conservation management and essentially reflect the future viability of wildlife populations [20, 27, 28], we hope that our results will benefit management practices and help to ensure a future for lions in the Lowveld of Zimbabwe.

## Materials and methods

### Laboratory procedures

The study was approved by the University of Johannesburg’s Ethical Committee. Samples were collected in Zimbabwe and taken to South Africa under import permit 13/1/1/30/2/0-2015/01/003889 and CITES permit ZW/0081/2015 & ZW/0082/2015. Samples were collected opportunistically when lions were fitted with radio-collars, removed from illegal snares, or found dead in the field. When an individual was found dead, a piece of tissue (approximately 5 by 5 cm) was clipped from the ear. When lions had to be immobilized for de-snaring or radio-collaring, 2 ml of blood was drawn from the leg. For immobilization, darts were fired from a maximum distance of 30 m (usually <25 m) to reduce the possibility of physical injury. Injury resulting from darting is very rare, and always minor, although dart wounds are routinely cleaned and flushed with antibiotics as a precaution. A combination of Medetomidine (Dormitor), a sedative in the Imidazole group (α-2 agonists) and Ketamine (a short-acting cyclohexylamine knock-down drug) was used as a sedative and analgesic. Blindfolds and ear plugs were used throughout the handling process to minimise any external stimuli.

A total of 42 samples were collected (25 from SVC; 10 from GNP; 7 from BVC; see S1 Table), and stored in 5 ml microtubes containing RNA stabilization solution kept at -20°C. In the laboratory, we extracted DNA using the QIAamp DNA Mini Kit (Qiagen) for blood and tissue samples, according to manufacturer’s instructions. All samples were genotyped for eleven polymorphic microsatellite loci (FCA031, FCA069, FCA075, FCA085, FCA096, FCA113, FCA126, FCA275, FCA310, FCA453, FCA506; see S2 Table for locus information) initially developed for the domestic cat *Felis catus* [29, 30]. The forward primer of each locus was labelled with a fluorescent dye (5HEX, 56-FAM, or 5ATTO550N) and amplified using the Qiagen Multiplex PCR mix. PCR thermocycling was performed with a hot-start at 95°C for 15 min, followed by 45 cycles of denaturation at 94°C for 30 sec, annealing at 62°C for 1.30 min, and extension at 72°C for 1 min, followed by a final extension step at 60°C for 30 min. We amplified and genotyped all DNA samples twice, and samples that showed weak amplification three times. Samples with missing data for three or more loci were excluded from analysis.

We conducted sequence-based genotyping for the mitochondrial cytochrome-*b* gene (primer combination described in [31]). The PCR reactions contained 1 unit of enzyme (SuperTherm; Southern Cross Biotechnology), 20 pmol (0.75 μM) of each primer, 1x PCR Buffer, 2.5 mM MgCl_2_, 200 μM dNTP and ~ 10 ng of DNA product. PCR thermocycling was performed with a hot-start at 95°C for 5 min, followed by 40 cycles of denaturation at 94°C for 45 sec, annealing at 50°C for 30 sec, and extension at 72°C for 45 sec, followed by a final extension step at 72°C for 10 min. Successful amplification was verified in 1% agarose gels. Amplicons were cycle sequenced using BigDye chemistry (Life Technologies), and analysed on an ABI 3730 DNA Analyzer (Life Technologies).

### Genetic analyses

Microsatellite loci were scored using Geneious 6.1.5 (Biomatters Ltd.) and tested for linkage disequilibrium (using log-likelihood ratio G-statistics) and deviation from Hardy-Weinberg equilibrium in FSTAT 2.9.3.2 [32]. We used MICRO-CHECKER version 2.2.3 [33] to detect genotyping errors in the form of null alleles, and individuals were genotyped twice to verify the accuracy of genetic profiles. Significance values were adjusted according to the Bonferroni correction. Allelic and private allelic richness were computed for each population in HP-Rare, following a rarefaction method to compensate for uneven sample sizes [34]. Gene diversity based on observed (*H*_*O*_) and expected (*H*_*E*_) heterozygosity, as well as genetic distances (F_ST_), was calculated using Arlequin 3.5 [35]. STRUCTURE 2.3.4 [36] was used to test for possible genetic clustering amongst the lion populations. We chose the admixture model for the ancestry of individuals and assumed correlated allele frequencies. The program was run for 1,000,000 MCMC repetitions, with a burnin period of 10%, and a number of 20 iterations. The most likely number of clusters (K) was determined by estimating LnP and ΔK using the Evanno method [37] as implemented in STRUCTURE HARVESTER [38]. CLUMPAK was used to combine the outputs from all the iterations [39]. Pairwise relatedness (*R*) was calculated for all individuals within each population using maximum likelihood estimates of relatedness calculated with ML-Relate [40].

For the mitochondrial data, chromatograms were checked using Geneious 6.1.5 (Biomatters Ltd.) and aligned using Clustal W as implemented in the Geneious software. Sequences were deposited in the GenBank database under accession numbers MG490402 (BVC1), MG490403 (BVC2) and MG490404 (SVC1). For the assessment of overall spatial structure we used NETWORK [41] to create a median-joining haplotype network. For comparison, we added published sequences from other lion populations [42, 43, 44, 45]; detailed information of the haplotypes used during this study can be found in S3 Table.

## Results

### Genetic variation

A total of 42 lions were genotyped successfully for 11 microsatellite loci (see S4 Table for generated microsatellite data). No deviations from Hardy-Weinberg equilibrium were found either for a locus-by-locus, or population-by-population, approach. None of the 11 loci were linked (linkage disequilibrium), as was also reported for the domestic cat [30]. The probability of null alleles across populations and loci was acceptably low (0.07; P < 0.05). Genetic diversity indices are presented in Table 1. On average, 3.5 alleles per locus are present in the SVC lions, while expected heterozygosity and observed heterozygosity are 0.41 and 0.38 respectively across all the loci. Four private alleles are present in this population. The SVC lions appear to be the genetically most depauperate population in the Lowveld region of Zimbabwe. By comparison, all diversity indices are higher in both the BVC and GNP, with BVC lions being characterized by *H*_*E*_ of 0.66, and GNP by an *H*_*E*_ of 0.53. Eleven private alleles are present in the BVC, and five in the GNP population. Inbreeding is also apparent in the SVC (F_IS_ = 0.171), yet is found to be higher in the BVC lion population (F_IS_ = 0.208).

**Table 1:** Genetic diversity indices, based on 11 microsatellite loci, of lions in the Savé Valley Conservancy (SVC), Bubye Valley Conservancy (BVC) and Gonarezhou National Park (GNP) in Zimbabwe. Data from other lion populations in Africa are added for comparisons; these were Ngorongoro Crater (NGR; Tanzania), Serengeti National Park (SER; Tanzania), Etosha National Park (ETO, Namibia); Kruger National Park (KRU, South Africa) [46], Pendjari National Park (PEN; Benin); Waza National Park (WAZ; Cameroon), Bénoué Ecosystem (BEN; Cameroon), Zakouma National Park (ZAK; Chad), Garamba National Park (GAR; DRC), Amboseli National Park (AMB; Kenya), Luangwa Valley (LUA; Zambia), Kalahari-Gemsbok National Park (GEM; South Africa), Kruger National Park (KGR; South Africa) [47].

Population	N_S_	N_L_	A	A_P_	H_O_	H_E_	F_IS_
**SVC**	25	11	3.5 (±1.1)	0.45	0.38 (±0.2)	0.41 (±0.2)	0.171
**BVC**	7	11	3.7 (±1.2)	0.63	0.53 (±0.2)	0.66 (±0.1)	0.208
**GNP**	10	11	3.7 (±1.1)	1.25	0.57 (±0.3)	0.53 (±0.2)	-0.085
**NGC**	10	88	2.9	-	0.43	0.40	-
**SER**	10	88	3.5	-	0.47	0.47	-
**ETO**	10	77	2.6	-	0.38	0.37	-
**KRU**	10	77	3.4	-	0.47	0.44	-
**PEN**	5	20	3.0	0.05	0.65	0.55	-0.204
**WAZ**	9	20	3.2	0.0	0.68	0.61	-0.129
**BEN**	3	20	2.9	0.05	0.58	0.61	0.060
**ZAK**	4	15	2.6	0.20	0.6	0.56	0.085
**GAR**	7	20	4.7	0.10	0.74	0.7	-0.066
**AMB**	7	20	2.7	0.0	0.51	0.5	-0.025
**LUA**	9	20	4.8	0.15	0.57	0.69	0.182
**GEM**	10	20	4.0	0.05	0.61	0.66	0.082
**KGR**	10	20	4.6	0.25	0.69	0.69	-0.002

N_S_ = sample size; N_L_ = number of microsatellite loci; A = average number of alleles per locus

A_P_ = private allelic richness; H_O_ = observed heterozygosity; H_E_ = expected heterozygosity; F_IS_ =

inbreeding coefficient;—indicates data not available.

This general trend (SVC having the lowest genetic diversity) held overall when allelic richness was estimated using the rarefaction method (to compensate for different samples sizes). The SVC population returned a value of 2.28, GNP lions 2.84, and the BVC population 3.29. When the rarefaction method was used to estimate private allelic richness [34], the SVC lions again are genetically most depauperate (A_P_ = 0.45) when compared to the GNP (A_P_ = 0.64) and BVC (A_P_ = 1.25). However, the number of alleles found in our study populations seem to be average when compared across Africa (see Table 1). When observed and expected heterozygosity were compared to other populations, values in the SVC are most comparable to lions in the Ngorongoro Crater and Hluhluwe-Emfolozi National Park, which both went to a population bottleneck [43, 47]. Inbreeding levels in the SVC and BVC lion populations are significantly high in comparison, and equivalent levels are only found in Luangwa Valley, Zambia [47].

Pairwise relatedness between SVC individuals of uncertain parentage is *R* = 0.14, similar to GNP lions (*R* = 0.13), which is most typical for third-order relatives (*R* = 0.125; [48, 49]). This level of relatedness is not uncommon in lions due to cooperative breeding and close kinship relations in prides [50]. Amongst 300 pairwise comparisons, we found 24 parent-offspring relationships, 11 full-sibling relationships, and 22 half-sibling relationships. The relatedness value amongst BVC lions is higher (with *R* = 0.18) and more typical for second-order relatives, which means there are generally more kin-linked individuals in this population.

**Fig 2:**
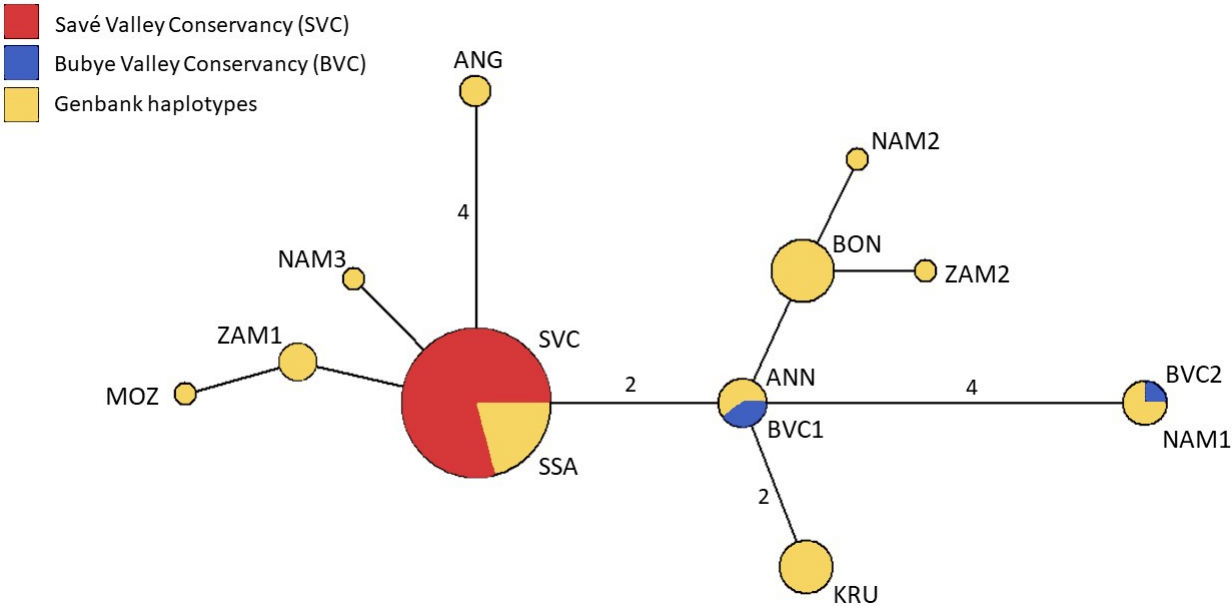
Median-joining network based on cytochrome-*b* haplotypes. Three lion populations in Zimbabwe, the Savé Valley Conservancy (SVC), Bubye Valley Conservancy (BVC) and Gonarezhou National Park (GNP), were compared with eleven haplotypes found in southern Africa: ANG (Angola), KRU (South Africa), ZAM1, ZAM2 (Zambia), NAM1, NAM2, NAM3 (Namibia), MOZ (Mozambique), ANN (Angola, Namibia), BON (Zimbabwe, Botswana, Namibia, South Africa), and SSA (Zimbabwe, Zambia, Botswana, Namibia, South Africa) [42, 43, 44, 45].

Only three mitochondrial DNA haplotypes, based on the 456 bp segment, are found for all 42 lions from the Zimbabwe Lowveld area. Two of these are unique to the BVC, and the other haplotype is shared amongst 35 individuals in the SVC and GNP, which makes the SVC and GNP populations completely monomorphic for the mitochondrial DNA segment (see Fig 2). It further suggests that the maternal lineage that founded the SVC and GNP populations are unrelated to the lineages that founded the current BVC population. We compared the haplotypes found here to those reported for lion populations Southern Africa (retrieved from Genbank; [42, 43, 44, 45]). The haplotype found in the SVC and GNP populations is very common among lions in southern Africa, and has previously been found in Namibia (the Erongo area), Botswana (Chobe NP), Zambia (i.e. Kafue and Luangwa NP), other parts of Zimbabwe (the Chitungwiza and Gweru area), and South Africa (e.g. Kruger NP and Hluhluwe-Umfolozi NP). The two haplotypes that are found in the BVC population are identical to haplotypes reported from Namibia ([45]). This confirms that the lions translocated to BVC were of Namibian origin, and explains their distinction from the SVC and GNP.

### Population differentiation

**Fig 3:**
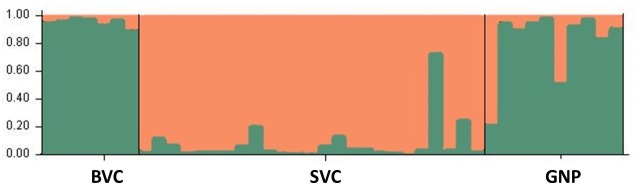
Structure plots for three lion populations in the Lowveld of Zimbabwe: Bubye Valley Conservancy (BVC), Savé Valley Conservancy (SVC), and Gonarezhou National Park (GNP). Two genetic clusters, corresponding to a green and red colour, are suggested (K = 2).

To test for geographic population structure, we compared the allele frequencies in the SVC lion populations with BVC and GNP. STRUCTURE analyses suggest K = 2 as the most likely number of genetic clusters (Ln P(K) = -857,6; ΔK = 887,9), in which the SVC clusters separately from the BVC and GNP populations (Fig 3). Very few individuals in the BVC and SVC populations show signs of admixture, while admixture is more evident in the GNP lions. An analyses of molecular variance indicates that 22% of the variation is accounted for by differences between populations (F_ST_ = 0.22; P < 0.001). There are some indications of population differentiation between the three lion populations in the region, with the highest genetic distance (as measured by F_ST_ values) found between the BVC and SVC (F_ST_ = 0.32) and the lowest between the BVC and GNP (F_ST_ = 0.12) (all values are significant at P < 0.001). The effect of inbreeding, and subsequent loss of alleles, is unlikely to have caused the genetic distinction of the SVC population, as the similarly inbred BVC population still clusters together with GNP lions. It could indicate that the SVC lions have been completely isolated during their recent population recovery, while the BVC and GNP lions still had some levels of population admixture.

## Discussion

Lions are one of the most iconic species on earth, but are experiencing significant declines in population numbers and available habitat and consequently have become vulnerable to the negative effects of genetic drift and inbreeding [4, 19]. One of the last remaining lion strongholds occurs in the Greater Limpopo Transfrontier conservation area, of which the Lowveld region of Zimbabwe forms part of. An understanding of population demography trends and connectivity amongst reserves or protected areas provide vital data to inform management.

### Genetic variability

The lion population in the Savé Valley Conservancy in Zimbabwe today numbers more than 200 individuals, but was founded by only approximately 15 individuals. The nearby Bubye Valley Conservancy contains a population of 500–550 lions, which originated from 13 translocated individuals. Reserves and protected areas in Zimbabwe are fenced, with increasing human settlements between reserves. Taken together, questions emerged regarding the genetic diversity of the lions in the SVC and BVC and the possible differentiation between lion populations in the area due to effects of genetic drift. Indeed, we confirmed the loss of allelic richness and low levels of heterozygosity [51–53]. When expected heterozygosity in the SVC population was compared to other lion populations in Africa, the level found in the SVC was comparable to the lion population in the Ngorongoro Crater, which is known to have suffered from a severe bottleneck [54]. When comparing inbreeding coefficients, the values found for the SVC and BVC related most to a population Zambia, although the high F_IS_ values in this population were considered to be caused by the Wahlund effect rather than a small number of founders [44]. A more comparable level of inbreeding (F_IS_ = 0.228) was found in Hluhluwe-Emfolozi National Park (before new individuals were translocated to the area), which is an isolated population that was founded by only a handful of lions [43]. Even though good management in the SVC and BVC led to the rapid recovery of lion numbers, preventing further losses of alleles, inbreeding is still of concern, as inbred lion populations are known to be more vulnerable to the impact of diseases and offtake by trophy hunting [54].

Relatedness of individuals in the SVC population (*R* = 0.14) was typical of second-order relatives, and this value was lower than the relatedness found in the Etosha lion population (*R* = 0.21; [53]). Interestingly, extra-group paternity (mating outside the social structure) occurred in the Etosha population, which lead to reduced relatedness amongst individuals [55]. It is possible that extra-group paternity, as a natural response to avoid inbreeding [56, 57], takes place in the SVC lion population. Extra-group paternity is rare in lions, but has been associated with low male densities [53], which may be driven by trophy hunting of males [11, 53]. However, trophy hunting also occurs in the BVC, yet relatedness is found to be higher in this population (R = 0.18).

The genetic erosion in the SVC and BVC lion populations is likely to progress in the future, due to the isolated nature of the reserves and limited space to allow for further population growth [19]. To maintain genetic diversity and avoid inbreeding in lions, a continuous population of at least 50 prides is recommended with no restrictions to dispersal [19]. In our study area, habitat loss and fragmentation keep lion numbers low and well below the 50 pride recommendation [10]. This raises concerns for the long-term survival of lion populations in the region, as reduced genetic variability is known to increase the extinction risk of wildlife populations [58–60]. For lions, negative impacts of genetic erosion have already been reported in the Ngorongoro Crater in Tanzania [54, 61] and the Hluhluwe-Umfolozi Park in South Africa [22].

### Genetic differentiation

The SVC lion population is genetically differentiated from the BVC and GNP populations. Although inbreeding and subsequent loss of alleles may cause populations to cluster as distinct lineages in cluster analysis [62], this does not seem to be the case here. The lion population in BVC shows higher levels of inbreeding compared with the SVC population, yet clusters with the GNP population. Genetic structure on such a small scale is less likely to occur when carnivores can cross fences [63]. For instance, no genetic differentiation was found in African wild dogs that occur alongside lions in the Lowveld of Zimbabwe [31]. Without the ability to cross fences, as is the case for lions, genetic drift will gradually lead to allelic differentiation between populations [64]. Genetic differentiation may also be driven, at least in part, by the genetic bottleneck and low number of founding individuals. Founder effect and genetic drift are major evolutionary forces that influence gene frequencies in isolated populations [65]. Our data confirm losses of alleles and shifts in the distribution of allele frequencies at neutral loci, which is known to be a result of population bottlenecks [66]. Allelic richness (A) and private allelic richness (A_P_) are also good predictors of population founding events [67], and these values are, indeed, estimated to be lower in the SVC population compared to GNP and BVC.

It is unlikely that the observed population structure is a result of the translocation of ten lions into the conservancy in 2005, which could have been the case if the immigrants were genetically divergent. However, private allelic richness is low in the SVC and cytochrome-*b* sequences suggest that GNP lions originate from the same historic population. The haplotype found in this area occurs across a wide range in southern Africa [45]. The mitochondrial heritage of the BVC population, on the other hand, relates to Namibia [45], which is the result of lion introductions in the late 1990s from a Namibian source population. After the re-establishment of the BVC population, dispersing lions also entered the park from surrounding areas such as Gonarezhou National Park.

### Conservation implications

From a conservation perspective, it is important to avoid loss of genetic variation in lion populations and maintain evolutionary potential required for future survival. A large population size and the stimulation of gene flow by dispersal is normally advised to achieve this [19–21, 28, 58, 59, 68], but the establishment of new habitat or corridors to link natural reserves are unrealistic under current circumstances in our study area [10]. As an alternative, translocation can be a useful conservation tool to overcome the threats posed by genetic erosion in the SVC and BVC [54, 69, 70]. Unrelated male lions could be introduced, while existing adults (≥5 years) are removed through trophy hunting [68]. Due to the strong founder effect in these areas, this will be the only way to supplement the resident lion populations with new genes. Genetic restoration through the introduction of unrelated individuals is known to increase genetic heterozygosity and improve survival and reproductive fitness in populations [22, 24]. Based on our results, lions in GNP would form a good source population when translocation is considered, as they originate from the same maternal lineage and are genetically distanced from the SVC lions.

## Supporting information

S1 Table Details of lion samples collected for this study. (XLSX)

S2 Table Overview of microsatellite loci used in this study. (XLSX)

S3 Table Overview of lion haplotypes used during this study, and primer details. (XLSX)

S4 Table Microsatellite data of all lions included in the data analysis. (XLSX)
